# Artificial Activation of *Escherichia coli mazEF* and *hipBA* Toxin–Antitoxin Systems by Antisense Peptide Nucleic Acids as an Antibacterial Strategy

**DOI:** 10.3389/fmicb.2018.02870

**Published:** 2018-11-26

**Authors:** Marcin Równicki, Tomasz Pieńko, Jakub Czarnecki, Monika Kolanowska, Dariusz Bartosik, Joanna Trylska

**Affiliations:** ^1^Centre of New Technologies, University of Warsaw, Warsaw, Poland; ^2^College of Inter-Faculty Individual Studies in Mathematics and Natural Sciences, University of Warsaw, Warsaw, Poland; ^3^Department of Drug Chemistry, Faculty of Pharmacy with the Laboratory Medicine Division, Medical University of Warsaw, Warsaw, Poland; ^4^Department of Bacterial Genetics, Institute of Microbiology, Faculty of Biology, University of Warsaw, Warsaw, Poland; ^5^Unit of Bacterial Genome Plasticity, Department of Genomes and Genetics, Pasteur Institute, Paris, France; ^6^Genomic Medicine, Medical University of Warsaw, Warsaw, Poland

**Keywords:** antimicrobial strategies, bacterial toxin–antitoxin systems, peptide nucleic acid (PNA), antisense oligonucleotides, *Escherichia coli mazEF* and *hipBA* targets

## Abstract

The search for new, non-standard targets is currently a high priority in the design of new antibacterial compounds. Bacterial toxin–antitoxin systems (TAs) are genetic modules that encode a toxin protein that causes growth arrest by interfering with essential cellular processes, and a cognate antitoxin, which neutralizes the toxin activity. TAs have no human analogs, are highly abundant in bacterial genomes, and therefore represent attractive alternative targets for antimicrobial drugs. This study demonstrates how artificial activation of *Escherichia coli mazEF* and *hipBA* toxin–antitoxin systems using sequence-specific antisense peptide nucleic acid oligomers is an innovative antibacterial strategy. The growth arrest observed in *E. coli* resulted from the inhibition of translation of the antitoxins by the antisense oligomers. Furthermore, two other targets, related to the activities of *mazEF* and *hipBA*, were identified as promising sites of action for antibacterials. These results show that TAs are susceptible to sequence-specific antisense agents and provide a proof-of-concept for their further exploitation in antimicrobial strategies.

## Introduction

Antibiotics are essential medicines used to prevent and treat otherwise incurable bacterial infections (Coates and Hu, 2007). However, the combination of widespread usage of antibiotics and bacterial evolution have decreased their efficacy, and stimulated the emergence of antibiotic-resistant bacteria (Fair and Tor, 2014). The traditional answer to this problem has been the introduction of new, improved versions of existing antibiotics that can kill the resistant bacterial mutants. However, simple molecular variants of commonly used antibiotics are becoming less effective in overcoming bacterial resistance mechanisms (Ventola, 2015a). The identification of new non-traditional targets for antimicrobials is therefore critical in combating infectious diseases caused by evolving pathogens (Ventola, 2015b).

Many bacteria contain toxin–antitoxin systems (TAs), usually composed of two genes that encode (i) a stable toxin that targets an essential cellular process, and (ii) a labile antitoxin that counteracts the activity of the toxin (Harms et al., 2018). These genetic modules are widely distributed in bacterial genomes, including clinical pathogens, but are not found in eukaryotes (Hayes and Van Melderen, 2011; Fernandez-Garcia et al., 2016; Lee and Lee, 2016). This makes them promising targets for the development of novel antibacterials (Kirkpatrick et al., 2016).

TAs have been divided into six classes, depending on the nature and the mode of action of the antitoxin molecule (Page and Peti, 2016). Type II TAs have been the most extensively characterized (Ma et al., 2015; Chan et al., 2016; Rocker and Meinhart, 2016). In this class, both the toxin and the antitoxin are proteins (Lobato-Márquez et al., 2016). Two particularly well studied type II TAs are *mazEF* (Hazan et al., 2004; Schuster et al., 2013; Tripathi et al., 2014) and *hipBA* (Schumacher et al., 2009; Germain et al., 2013; Kaspy et al., 2013). In the *mazEF* TAs family, the MazF toxin is an endoribonuclease, which cleaves mRNA at three-, five-, or seven-base recognition sequences in different bacteria resulting in translation inhibition (Yamaguchi and Inouye, 2009; Yamaguchi et al., 2012; Schifano et al., 2016). This cleavage occurs independently of both the ribosome and translation, and it is inhibited by the MazE antitoxin (Kędzierska and Hayes, 2016). In the *hipBA* TAs family, the HipA toxin targets the glutamyl-tRNA synthetase (GltX) involved in tRNA synthesis (Germain et al., 2013). The overproduction of HipA affects the integrity of *E. coli* cells and induces their lysis (Kędzierska and Hayes, 2016). The HipA toxin is neutralized by forming a complex with the HipB antitoxin (Wen et al., 2014). The *mazEF* and the *hipBA* loci are present in the chromosomes of many bacteria (Fu et al., 2009; Rothenbacher et al., 2012; Fernandez-Garcia et al., 2016), but their function has mainly been studied in *E. coli* (Hazan et al., 2004; Schumacher et al., 2009).

**FIGURE 1:**
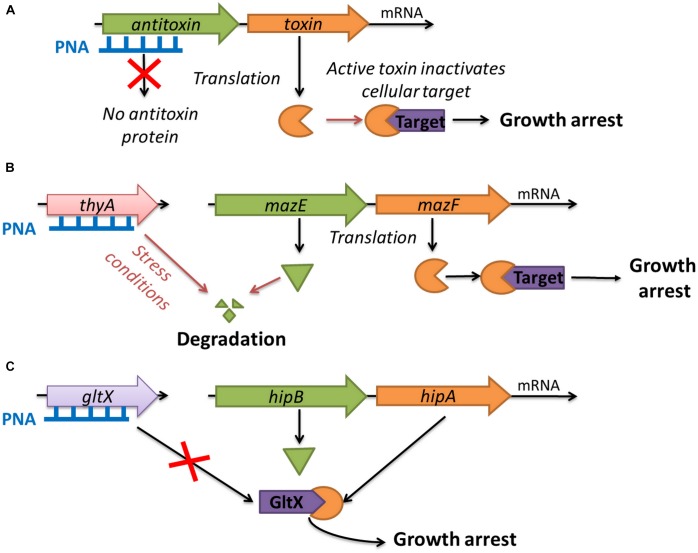
Strategies for using toxin–antitoxin systems as a target for antibacterials: **(A)** artificial activation of MazF and HipA toxins by antisense PNA targeted at the antitoxin genes; **(B)** indirect activation of MazF toxin by inducing thymine starvation through silencing of the *thyA* gene; **(C)** blocking the expression of the *gltX* gene which mimics the action of the HipA toxin.

The artificial activation of TAs has potential as an antimicrobial strategy (Lioy et al., 2010; Shapiro, 2013; Verma et al., 2015; Kim et al., 2018). To test this concept we have used antisense oligonucleotides to try to alter TAs activity and arrest the growth of bacteria (Figure 1). Antisense oligonucleotides bind to nucleic acids in a sequence-specific manner by Watson-Crick base pairing, and can affect the function of targeted mRNAs and silence genes (Dias and Stein, 2002). Due to the rapid degradation of natural oligonucleotides in the intracellular environment, chemically modified oligonucleotides, such as peptide nucleic acids (PNA), have been successfully used as silencing agents (Rasmussen et al., 2007). PNA is a DNA analog with a pseudo-peptide backbone composed of repeating *N*-(2-aminoethyl) glycine units linked by amide bonds (Nielsen et al., 1991). PNA oligomers show enhanced nuclease and protease resistance, improved binding affinity with natural nucleic acids, and negligible toxicity to eukaryotic cells (Good et al., 2001). Typically, antibacterial PNAs have been targeted against mRNAs of essential genes (Good et al., 2001; Nekhotiaeva et al., 2004; Patenge et al., 2013) or functional ribosomal sites (Hatamoto et al., 2009; Górska et al., 2016; Kulik et al., 2017). Earlier problems with the delivery of PNAs to bacterial cells have been solved by conjugating them to various carriers, such as cell penetrating peptides (CPP) (Abushahba et al., 2016) or vitamin B_12_ (Równicki et al., 2017).

In this study, we have investigated three innovative strategies related to the use of TAs and associated proteins as targets for antibacterial molecules (Figure 1). To the best of our knowledge, such approaches have not been reported so far. The first strategy was the artificial activation of the *E. coli* MazF and HipA toxins by using antisense PNA oligomers to inhibit the translation of the corresponding antitoxins (MazE and HipB, respectively, Figure 1A). The second strategy involved the indirect activation of the MazF toxin by inducing thymine starvation. This was achieved by silencing the *thyA* gene encoding thymidylate synthase, an enzyme involved in folic acid metabolism, which has been shown to interfere with *mazEF*-mediated growth inhibition (Figure 1B) (Engelberg-Kulka et al., 2005). The third strategy was to mimic the action of the HipA toxin by silencing the *gltX* gene encoding its cellular target glutamyl-tRNA synthase (Figure 1C).

To test these novel strategies, we first designed and synthesized antisense PNAs and evaluated their antimicrobial effectiveness by determining the minimal inhibitory concentration (MIC) for three strains of *E. coli*: a K-12 wild-type strain, the pathogenic strain O157:H7 (Łoś et al., 2008) and the extended spectrum β-lactamase-producing strain WR3551/98 (Baraniak, 2002) (Supplementary Table 1). Second, we examined the decay of the target mRNAs after treatment with different PNA concentrations, using reverse transcription real-time quantitative PCR (RT-qPCR). Third, we checked for any synergistic interactions between the *mazEF*-targeted PNAs and selected antibiotics (trimethoprim and sulfamethoxazole) by determining the fractional inhibitory concentration (FIC). Finally, we assessed the cytotoxicity of the oligonucleotides to eukaryotic cells in an assay using human embryonic kidney-293 cells (HEK-293).

## Results

### Selection of Targets for PNA Oligomers

Based on the predicted secondary and tertiary structures of the targeted mRNAs, four antisense PNA sequences were designed (Table 1). Their complementary sequences overlapped the most favorable mRNA sites that covered the translation start codon (Dryselius et al., 2003; Deere et al., 2005) and lacked stable secondary structures such as stem-loops or hairpins (Rasmussen et al., 2007).

**Table 1:** PNAs used in this study.

TA system	Name	Target	Sequence
			(N_term_–C_term_)^∗^
*mazEF*	anti-*mazE* PNA	*mazE*	(KFF)_3_K-cataaccctttc
	anti-*thyA* PNA	*thyA*	(KFF)_3_K-tcatggttcctc
*hipBA*	anti-*hipB* PNA	*hipB*	(KFF)_3_K-catgtcatacg
	anti-*gltX* PNA	*gltX*	(KFF)_3_K-gattttcatg
-	PNAnc	Control	(KFF)_3_K-tccattgtctgc
		non-complementary sequence	


*To increase water solubility, each PNA has a lysine residue at the C-terminus. ^∗^lower case letters indicate the PNA sequence.*

To validate the sequence homology of the PNA target sites in *E. coli* O157:H7 and WR3551/98, these regions were amplified by PCR using specific primers (Supplementary Table 2) and sequenced. All sequences were identical in the three *E. coli* strains, excluding any possibility that mutation of the mRNA target sites for the PNAs might cause decreased susceptibility. To deliver the PNAs into cells, we used a cell-penetrating peptide (KFF)_3_K, which was covalently linked to each PNA oligomer via an AEEA (aminoethoxyethoxyacetic acid) linker. (KFF)_3_K is the most widely used cell-penetrating peptide for achieving PNA entry into *E. coli* cells (Eriksson et al., 2002; Bendifallah et al., 2006).

As a negative control, a random PNA (PNAnc) unrelated to the target sequences was used (Table 1). See Materials and Methods for detailed synthesis protocols.

### *Escherichia coli* Growth Inhibition by Sequence-Specific PNAs

To evaluate the potential of antisense PNAs to inhibit the growth of *E. coli* strains, the minimal inhibitory concentrations (MIC) of the compounds were determined. All tested PNAs inhibited *E. coli* growth. First, the MICs for the (KFF)_3_K-PNAs targeted to antitoxins were measured.

**FIGURE 2:**
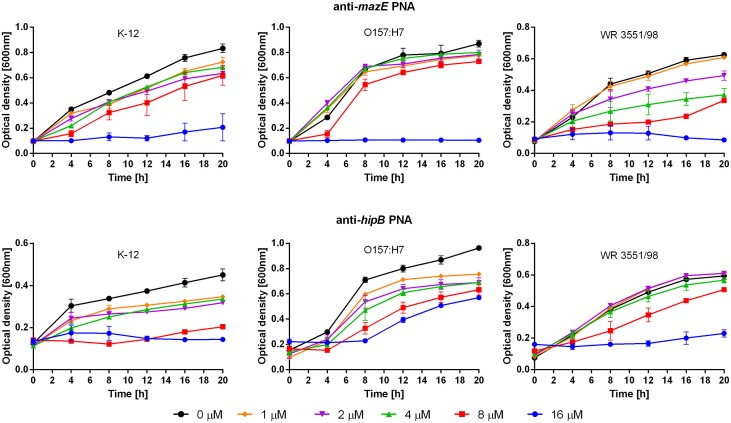
Growth inhibition after treatment with the anti-*mazE* (top) and anti-*hipB* (bottom) PNAs. Error bars represent the mean ± SEM, *n* = 3. For all strains the differences between 0 and 16 μM concentrations at 20 h are statistically significant with *P* < 0.001.

The MIC of the anti-*mazE* PNA was 16 μM for all three *E. coli* strains (Figure 2, top), while the anti-*hipB* PNA prevented the growth of *E. coli* K-12 at a concentration of 8 μM and *E. coli* WR3551/98 at 16 μM (Figure 2, bottom). In *E. coli* O157:H7, the antimicrobial activity of anti-*hipB* PNA was observed only up to 8 h of exposure (Figure 2, bottom). Therefore, the MIC was recorded as being >16 μM.

**FIGURE 3:**
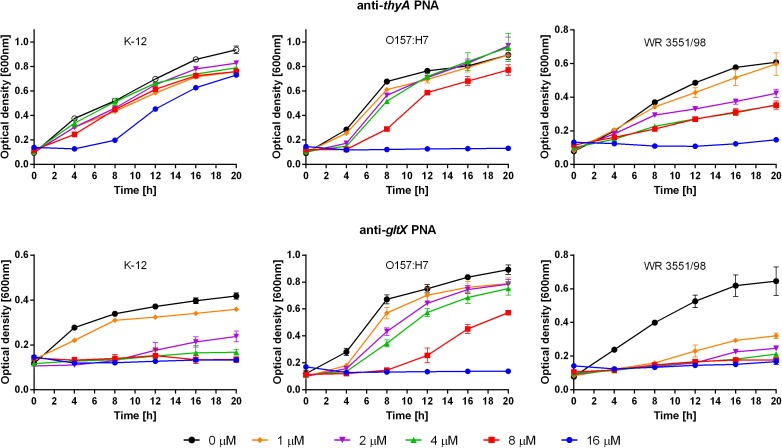
Growth inhibition after treatment with the anti-*thyA* (top) and anti-*gltX* (bottom) PNAs. Error bars represent the mean ± SEM, *n* = 3. For all strains the differences between 0 and 16 μM concentration at 20 h are statistically significant with *P* < 0.001.

Next, the antisense PNAs to the two TAs-associated target genes were tested (Table 1). The anti-*thyA* PNA had lower activity against *E. coli* K-12 (MIC > 16 μM) than against the strains O157:H7 and WR3551/98 (MIC = 16 μM) (Figure 3, top). After treatment with anti-*gltX* PNA, the growth of *E. coli* K-12, O157:H7, and WR3551/98 was prevented at MICs of 4, 16, and 1 μM, respectively (Figure 3, bottom).

To control for specificity of the interaction of the sequence-specific PNAs, the non-complementary PNAnc was tested (Table 1). No growth inhibition was observed following the addition of the (KFF)_3_K-PNAnc at concentrations of up to 16 μM (Supplementary Figure 1A). Addition of the (KFF)_3_K peptide alone at concentrations above 16 μM also failed to cause any significant reduction in *E. coli* growth (Supplementary Figure 1B).

To confirm that the observed growth inhibition was a consequence of toxin action, we used *E. coli* mutants lacking either the *mazF* (K-12 MG1655 *ΔmazF*) or *hipA* (JW1500-2 *ΔhipA*) toxin genes (Supplementary Table 1). The *ΔmazF* mutant was treated with anti-*mazE* PNA and the *ΔhipA* mutant with anti-*hipB* PNA. No growth inhibition of these mutant strains was observed following treatment with the corresponding PNAs at concentrations of up to 16 μM (Supplementary Figure 2). This result verified that the activity of the MazF and HipA toxins was the main cause of growth inhibition after treatment with the specific PNAs.

### Effect of PNAs on Corresponding Gene Transcripts

**FIGURE 4:**
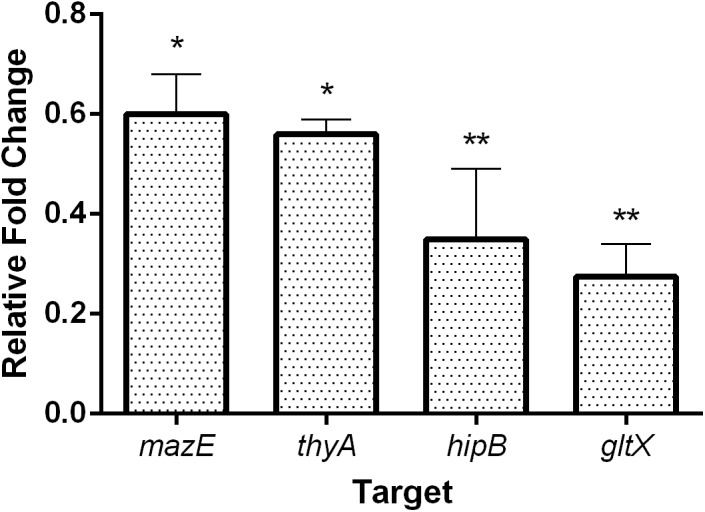
The effect of PNA treatment on the abundance of the targeted mRNA transcripts in *E. coli* K-12. The graph shows the relative expression fold change compared to respective untreated control samples. Error bars represent the mean ± SEM, *n* = 2. ^∗^*P* < 0.1, ^∗∗^*P* < 0.05.

We further tested whether the reduction in growth of *E. coli* upon treatment with sequence-specific PNAs was reflected in the changes of the corresponding gene transcript levels. To determine the level of the targeted mRNAs, the quantitative reverse transcription polymerase chain reaction (qRT-PCR) was used (Figure 4). *E. coli* K-12 cultures were treated with sub-MIC concentrations of each respective PNA and the level of mRNAs was compared to untreated control samples. Upon treatment with 8 μM anti-*mazE* PNA, the amount of *mazE* transcript was reduced to 60% (Figure 4). The level of *thyA* mRNA was reduced to 55% after treatment with 16 μM anti-*thyA* PNA. Similarly, the level of *hipB* and *gltX* transcripts was diminished to 40 and 35%, respectively, after treatment with sub-MIC concentrations (4 μM anti-*hipB* and 1 μM anti-*gltX*) of corresponding PNAs (Figure 4). We also monitored the mRNA levels of the *mazF* and *hipA* toxin genes. Treatment with PNA conjugates targeting these antitoxin genes did not influence the relative *mazF* and *hipA* mRNA levels detected by qRT-PCR (Supplementary Figure 3).

### Antimicrobial Synergy Between PNAs and Antibiotics

**FIGURE 5:**
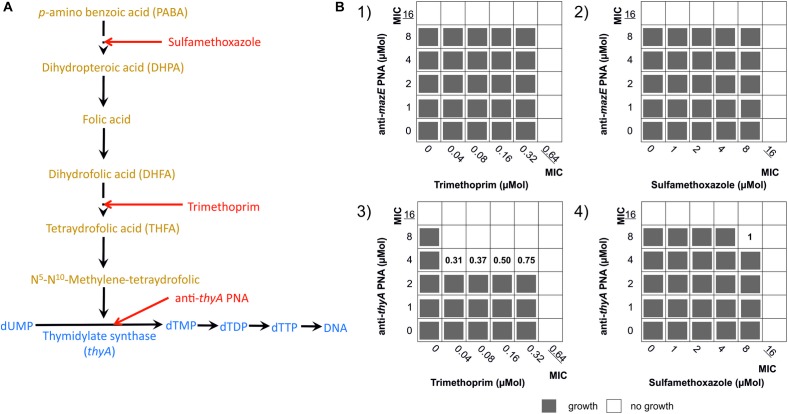
Effect of combining treatments that interfere with folic acid metabolism on the growth of *E. coli* O157:H7. **(A)** The main steps in the metabolism of folic acid (orange) and thymine (blue). Red arrows mark the points of action of sulfamethoxazole, trimethoprim, and (KFF)_3_K-PNA anti-*thyA*. Based on Engelberg-Kulka et al. (2004). **(B)** Checkerboard tests showing the FIC indices calculated for the combinations of **(B1)** trimethoprim/anti-*mazE* PNA, **(B2)** sulfamethoxazole/anti-*mazE* PNA, **(B3)** trimethoprim/anti-*thyA* PNA, and **(B4)** sulfamethoxazole/anti-*thyA* PNA. Results evaluated after 12 h of incubation.

It has been demonstrated that the antibiotics trimethoprim and sulfonamide induce thymine starvation by inhibiting folic acid metabolism, and this triggers *mazEF*-mediated cell death in *E. coli* (Engelberg-Kulka et al., 2004). Because folic acid metabolism inhibitors and the *mazEF*-targeted PNAs (anti-*mazE* and anti-*thyA*, Table 1) have related targets (Figure 5A), we sought to determine whether they act synergistically against *E. coli* O157:H7. The combinatory effects of antibiotics and PNAs were examined in checkerboard assays and quantified by the calculation of FIC indices. Following this protocol, the MIC for each compound was determined independently, and subsequently this was repeated for each concentration of the PNA combined with each concentration of the antibiotic (Figure 5B). The combinations of anti-*mazE* PNA/trimethoprim (Figure 5B1) and PNAs/sulfamethoxazole (Figures 5B2, B4) presented an interaction of indifference. However, the combination of anti-*thyA* PNA with trimethoprim resulted in a highly synergistic interaction (FICi = 0.31; Figure 5B3).

### Influence of PNAs on Human Cells

**FIGURE 6:**
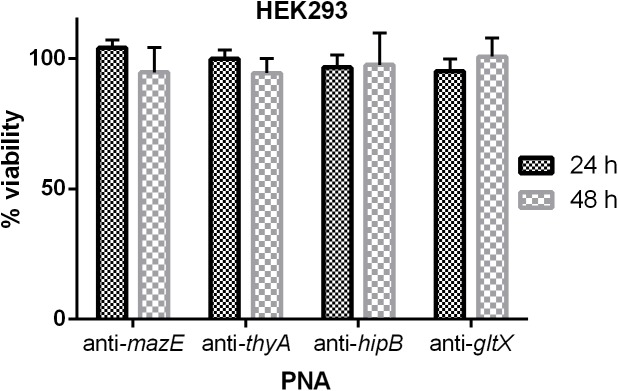
The cytotoxic effect of PNAs at a concentration of 32 μM on HEK-293 cells. The results are presented as the % viability in comparison to untreated control cells. Error bars represent the mean ± SD; *n* = 3.

The cytotoxic effect of the tested (KFF)_3_K-PNA conjugates on human embryonic kidney cells 293 (HEK-293) was evaluated. For these experiments the (KFF)_3_K-PNA conjugates were applied at a concentration of 32 μM. No significant impact on cell viability was observed after 24 or 48 h of treatment compared to untreated control cells (Figure 6).

## Discussion

The exploitation of TAs in antibacterial strategies via artificial activation of the toxin has been proposed (Shapiro, 2013; Williams and Hergenrother, 2014; Chan et al., 2015), but there have been no experimental data to support this idea until now. The results of this study provide a proof-of-concept for the use of bacterial TAs as novel targets for antimicrobial compounds. Operons encoding the targeted TAs, *mazEF* and *hipBA*, conserved in both laboratory and pathogenic *E. coli* strains (Fernandez-Garcia et al., 2016), were found to be promising candidates for antisense-based antibacterials. A reduction in *E. coli* growth was obtained by inhibiting the expression of the *mazE* and *hipB* antitoxins using PNA oligomers targeting their mRNAs.

To the best of our knowledge, artificial activation of toxins by antisense oligonucleotides has never been attempted as an antibacterial strategy (Narenji et al., 2017). PNAs have been tested as antibacterials, typically by targeting the expression of essential genes. The TAs genes are non-essential, and this might explain why the MICs obtained in this study are slightly higher than those previously reported for PNAs (Dryselius et al., 2003, 2005; Nikravesh et al., 2007). On the other hand, these MIC values are comparable with those reported for cases where essential sites on ribosomal RNA were targeted (Hatamoto et al., 2009; Górska et al., 2016; Kulik et al., 2017).

Furthermore, we tested PNAs specific for two additional targets related to the activities of *mazEF* and *hipBA*. First, we targeted the *thyA* gene in order to induce thymine starvation and as a consequence indirectly activate the MazF toxin. The other target was the *gltX* gene encoding glutamyl-tRNA synthase, the enzyme inhibited by HipA toxin. The assumption was that lowering the concentration of GltX should result in fatal effects for the bacterial cell, similar to those observed in the case of the HipA-mediated inactivation. Both approaches were successful, showing that multiple antimicrobial strategies might be based on the action of TAs.

With the PNAs targeted to inhibit the translation of the *thyA* and *gltX* transcripts, *E. coli* WR3551/98 turned out to be the most susceptible strain. This observation is consistent with a previous report that extended-spectrum beta-lactamase (ESBL)-producing *E. coli* strains are more susceptible to an anti-*rpoD* PNA than the wild-type strain (Bai et al., 2012). Furthermore, the obtained MICs for the anti-*thyA* and anti-*gltX* PNAs are generally comparable with those reported for PNAs targeting essential genes. For example, in the studies employing *E. coli* K-12, the MIC of a PNA targeting the *acpP* gene was 1 μM (Nikravesh et al., 2007), while those of PNAs against the *fabI* and *fabD* genes were both 3 μM (Dryselius et al., 2005). Overall, *E. coli* O157:H7 was the least susceptible to the PNAs tested in this study. This observation may be due to the diverse genetic background of the *E. coli* strains and consequently different expression levels of the target genes in response to respective PNAs. Another possible reason could be due to the differences in the structure of the lipopolysaccharide in *E. coli* strains, which is a known limiting factor for antimicrobial PNA action (Good et al., 2000).

Antisense silencing using PNA in bacteria leads to translational repression via steric blocking of the ribosome assembly on the mRNA (Rasmussen et al., 2007). As a consequence, such non-translated transcripts are degraded (Dryselius et al., 2006; Goh et al., 2009). Therefore, the effectiveness of antisense silencing can be estimated by quantifying mRNA abundance (Kurupati et al., 2007). Note, that the qRT-PCR experiments were performed on the *E. coli* K-12 strain and not on O157:H7 or WR3551/98 strains. This was mostly because the yield and quality of the RNA (which are critical in qRT-PCR analysis), isolated from *E. coli* O157:H7, were much lower than the RNA isolated form *E. coli* K-12. Although there are differences in the response of the tested *E. coli* strains to different PNA oligomers, the tendency is similar in the O157:H7 and WR3551/98 strains as compared to K-12. We thus used wild-type *E. coli* K-12 as a background in all qRT-PCR experiments.

We detected a significant reduction in the levels of the targeted mRNAs upon treatment with the complementary PNAs (Figure 4), confirming their sequence-specific binding to these transcripts. Similar observations have been reported in various bacterial species, e.g., in *Streptococcus pyogenes* where the application of anti-*gyrA* PNA decreased the *gyrA* mRNA level to about 50% of the untreated control value (Patenge et al., 2013). In *E. coli* the level of *acpP* mRNA was diminished to about 60% of the control following treatment with anti-*acpP* PNA (Nikravesh et al., 2007). To demonstrate the absence of off-target activity of the tested PNAs, the levels of the mRNAs for the *mazF* and *hipA* toxin genes were monitored after treatment with PNA conjugates targeting the corresponding antitoxin genes. The transcript levels of these toxin genes remained unchanged as compared to the untreated control samples (Supplementary Figure 3). Since the antitoxin and toxin genes are organized in operons, we presume that disruption of translation of the former does not totally prevent translation of the latter. Although the genes are co-transcribed as a single mRNA, the toxin gene carries its own ribosome binding site located within the upstream antitoxin gene (Chan et al., 2012).

The use of synergistic antibiotic/non-antibiotic combinations is a promising option for maximizing the therapeutic effect against problematic drug-resistant pathogens (Schneider et al., 2017). Moreover, the combination of agents makes the emergence of resistance in bacteria less likely (Doern, 2014). The *in vitro* effectiveness of the combined application of antisense PNAs and traditional antibiotics has already been demonstrated for several bacterial pathogens such as *Campylobacter jejuni* (Mu et al., 2013), *Streptococcus pyogenes* (Patenge et al., 2013), *Staphylococcus aureus*, and *E. coli* (Dryselius et al., 2005). However, in all these reports, the authors described synergistic antimicrobial effects for the combinations of drugs sharing the same targets. In addition, Patenge et al. (2013) also showed synergy between agents independent of the targeted pathways.

The combination of trimethoprim and sulfamethoxazole (co-trimoxazole) is commonly used in clinical treatment because of their impedance of two sequential steps in folic acid synthesis (Figure 5A) (Bushby, 1975; Harvey, 1982). Since both agents trigger *mazEF*-mediated growth inhibition (Engelberg-Kulka et al., 2004), synergistic interactions with the *mazEF*-targeted PNAs were expected. However, we found strong synergy only for the combination of anti-*thyA* PNA and trimethoprim (Figure 5B3). The combination of the same anti-*thyA* PNA and sulfamethoxazole resulted in an interaction of indifference (Figure 5B4). This observation is interesting with regard to the mechanism of synergy. All three agents act on the same metabolic pathway (Figure 5A), but the synergistic effect was observed only for two combinations, i.e., trimethoprim/sulfamethoxazole and trimethoprim/anti-*thy* PNA. Dryselius et al. (2005) tested combinations of PNAs and drugs against the folate biosynthesis pathway in *E. coli* and *S. aureus*. Surprisingly, they found synergy when sulfamethoxazole, which targets the dihydropteroate synthase (FolP), was combined with anti-*folP* PNA. However, they did not observe synergy when the anti-*folP* PNA was combined with trimethoprim, which targets dihydrofolate reductase (FolA). Similarly, the combination of anti-*folA* PNA and trimethoprim showed synergy but the sulfamethoxazole/anti-*folA* PNA combination showed indifference (Dryselius et al., 2005). Further investigation is required to understand these drug interactions, but the results of this and previous studies show how combinations of PNAs and antibiotics can be applied to help decipher mechanisms of drug action.

PNA oligonucleotides are generally considered to have negligible toxicity for eukaryotic cells (Good et al., 2001; Evers et al., 2015). However, whether or not PNAs elicit toxic effects is highly dependent on their sequence composition and the presence of a transporter vehicle (Pandey et al., 2009; Rozners, 2012; Zeng et al., 2016). We demonstrated that treatment of HEK-293 cells with the designed PNAs at a concentration of 32 μM did not cause significant cytotoxicity. This observation is consistent with those reported previously (Sharma et al., 2017). However, another study found that 2-aminopyridine-modified PNAs and their peptide conjugates were not cytotoxic up to 10 μM, but toxicity occurred at higher concentrations (Hnedzko et al., 2017). For this reason, confirmation that PNA-peptide conjugates are not toxic to mammalian cells is crucial before their therapeutic use is considered.

Two potential undesirable effects of artificial activation of TAs are bacterial persistence and biofilm formation (Lewis, 2007; Díaz-Orejas et al., 2017). The precise mechanism of persister formation is currently unknown (Formation et al., 2017), but according to previous reports, only overexpression of the toxin induces persistence (Pedersen et al., 2002; Lewis, 2008; Harrison et al., 2009; Wood et al., 2013). The PNAs employed in the present study did not lead to overexpression of the toxin (Supplementary Figure 3), so we speculate that the risk of dormant cell formation is relatively low. On the other hand, there is evidence that toxin–antitoxin modules are not the only factors that contribute to the formation of persister cells (Fisher et al., 2017). The deletion of multiple TAs reduces persisters but does not fully abolish their presence (Kint et al., 2012; Chowdhury et al., 2016). It is possible that there is heterogeneity within the persister population, with several different pathways contributing to the formation of persister cells. Furthermore, we speculate that the artificial activation of *E. coli* TAs by antisense PNA oligomers could induce the stringent response. This phenomenon is a bacterial response to stress conditions, e.g., amino-acid starvation (Cashel et al., 1996). The stringent response has been described as an important mechanism for the persister formation as a reaction to a diauxic growth (Amato and Brynildsen, 2015). However, how the formulation of persister cells is connected to this stress response is still unknown. Persister cells are present in each population prior to any treatment with antibacterial agent. Therefore, further investigation is required to determine the exact roles of central metabolism and toxin–antitoxin modules in the formation of persisters. In the future, we plan to determine whether artificial activation of TAs by PNAs influences persister formation and if it triggers the stringent response.

## Materials and Methods

### Target Site Selection

The genome sequence of *E. coli* K-12 was obtained from the KEGG database (Ogata et al., 1999). For secondary and tertiary structure prediction of the targeted mRNAs, the Mfold (Zuker, 2003), and RNAComposer (Popenda et al., 2012) software were used, respectively. To ensure target specificity of PNAs, the target sites were examined for uniqueness within the *E. coli* K-12 genome using the on-line search tools GenoList (Lechat et al., 2008) and RiboScanner (Górska et al., 2016), with special consideration given to the start codon region that includes the consensus Shine-Dalgarno sequence.

### Reagents and Conditions for (KFF)_3_K-AEEA-PNA Synthesis

Commercial reagents and solvents were used as received from the supplier. NovaSyn^®^TG Sieber resin for PNA synthesis was obtained from Merck and Fmoc/Bhoc-protected PNA monomers (Fmoc-PNA-A(Bhoc)-OH, Fmoc-PNA-G(Bhoc)-OH, Fmoc-PNA-C(Bhoc)-OH, Fmoc-PNA-T-OH) from Panagene and Link Technologies Ltd. Fmoc-AEEA-OH was purchased from Link Technologies Ltd. Nα-Fmoc protected L-amino acids (Fmoc-Lys(Boc)-OH, Fmoc-Phe-OH) were obtained from Sigma-Aldrich. Preparative chromatography was performed using C18 reversed-phase silica gel 90 Å (Sigma-Aldrich) with redistilled water and HPLC grade MeCN as eluents with the addition of HPLC grade trifluoroacetic acid (TFA) (0.1% v/v solution). The following conditions were used for HPLC: column – Eurospher II 100–5 C18, 250 mm Å – 4.6 mm with a precolumn; pressure – 230 bar; flow rate – 4.5 ml/min; room temperature; detection – UV/Vis at a wavelength of 267 nm.

### Synthesis of (KFF)_3_K-AEEA-PNA Conjugates

The conjugates of (KFF)_3_K with PNA connected via an aminoethylethanolamine linker (AEEA) were in-house synthesized manually using the Fmoc solid-phase methodology at 10 μmol scale. The syntheses were conducted using 9-Fmoc-amino-xanthen-3-yloxy TG resin with amine group loading of 190 μmol/g. This resin carries a linker, which yields a C-terminal amide upon TFA cleavage of the PNA. In all syntheses, Lys was the first monomer attached to the resin. PNA oligomers were synthesized using a 2.5-fold molar excess of the Fmoc/Bhoc-protected PNA monomers. To activate the monomers, we used a 2-(1H-7-azabenzotriazole-1-yl)-1,1,3,3-tetramethyluronium hexafluorophosphate (HATU), *N*-methylmorpholine (NMM), and 2,6-lutidine (0.7:1:1.5) mixture in DMF/NMP (1:1, v/v) solution. Coupling of the activated compounds was carried out two times for 30 min each. The Fmoc group was removed with 20% (v/v) piperidine in DMF (2 mm × 2 min). After the PNA oligomer synthesis and N-terminal Fmoc deprotection, Fmoc-AEEA-OH was attached to the N-terminus. Then, the N-terminal Fmoc was removed, and amino acids were coupled to yield the (KFF)_3_K peptide. We used a 3-fold and 1.5-fold molar excess of Fmoc-AEEA-OH and the Fmoc-protected amino acids (Fmoc-Lys-(Boc)-OH, Fmoc-Phe-OH) in the respective couplings performed two times for each substrate (for 60 and 40 min, respectively). The reagents were activated with HATU and the addition of HOAt and collidine (1:1:2) and *N*,*N*-dimethylpyridin-4-amine (DMAP) as a catalyst, in DMF/NMP (1:1, v/v) solution. The Fmoc groups of Fmoc-AEEA-OH and amino acids were deprotected using 20% (v/v) piperidine in DMF in two cycles (5 and 15 min). All of the protecting groups were removed and cleavage of the (KFF)_3_K-AEEA-PNA conjugates from the resin was accomplished by treatment with a TFA/triisopropylsilane/m-cresol (95:2.5:2.5; v/v/v) mixture for 60 min. The crude products were precipitated with cold diethyl ether, collected by centrifugation, decanted, lyophilized, and subsequently purified by RP-HPLC. The HPLC methods and molecular masses are shown in Supplementary Table 3, and the RP-HPLC chromatograms and MS spectra of the purified products in Supplementary Figures 4–8.

### Bacterial Strains and Growth Conditions

The strains used in this study are listed in Supplementary Table 1. All strains were grown in LB or LA medium at 37°C. When necessary, the media were supplemented with kanamycin (50 μg/ml), chloramphenicol (20 μg/ml), sucrose (10% w/v), or diaminopimelic acid (DAP, 0.3 mM). For susceptibility tests, the strains were grown in cation-adjusted Mueller Hinton Broth (Difco) at 37°C with shaking.

### PCR for Sequencing

Bacterial gDNA was extracted from cell pellets using a Bacterial & Yeast Genomic DNA Purification Kit (EURx, Poland) according to the manufacturer’s instructions. Based on the *E. coli* K-12 MG1655 genome sequence (RefSeq assembly accession: GCF_000005845.2) appropriate oligonucleotide primers were designed (Supplementary Table 2) for the amplification of fragments of the genes overlapping the targeted sequences. The PCR products were purified and sequenced.

### Genetic Manipulations and Strain Construction

DNA manipulations were performed using standard procedures, and according to the manufacturer’s instructions included in kits for DNA isolation and PCR (Phusion, Thermo Fisher Scientific). Deletion of the *E. coli* K-12 MG1655 *mazF* gene was performed using the gene replacement method (Pósfai et al., 1999). The plasmid for the mutagenesis [pDS132-mazFKm was generated by the Gibson assembly procedure (Gibson et al., 2009)]. The DNA fragments used for the assembly were amplified by PCR using the following primer pairs: (i) mazF1/mazF2 and mazF3/mazF4 – amplification of DNA fragments flanking the *mazF* gene using MG1655 genomic DNA as template, (ii) KML/KMR – amplification of the kanamycin resistance gene from plasmid pMSB1 (Dziewit et al., 2011), and (iii) pds132X/pds132Y – amplification of mobilizable vector pDS132 carrying the *sacB* gene enabling counter-selection with sucrose (Philippe et al., 2004). The assembled plasmid was transformed into *E. coli* DH5αλpir cells, carrying the *pir* gene required for pDS132 replication. Isolated pDS132-mazFKm plasmid DNA was then used to transform *E. coli* strain β2163 (carrying the *pir* gene) and a single transformant was used as a donor in a bi-parental mating with strain MG1655 (without the *pir* gene) as the recipient. The *ΔmazF* mutant colonies (in which the *mazF* gene was replaced by the kanamycin resistance gene following homologous recombination between the flanking regions) were selected on LA medium containing kanamycin and sucrose. The deletion was confirmed by PCR using primers LmazFspr/RmazFspr and sequencing of the amplified DNA fragment.

### Minimal Inhibitory Concentrations

The minimal inhibitory concentrations (MICs) of the antibiotics, (KFF)_3_K-PNAs and free (KFF)_3_K were determined by broth microdilution according to Clinical and Laboratory Standards Institute method M07-A10 (CLSI, 2015). Each MIC was scored as the lowest concentration that prevented visibly detectable growth. Cells were cultured in Mueller-Hinton Broth (MHB) to exponential phase and diluted to ∼5 × 10^5^ CFU/ml. Aliquots of the cell suspensions were added to the wells of sterile 96-well plates containing dilutions of the tested agents and these plates were incubated at 37°C in a microplate reader for 20 h. During incubation, the optical density at 600 nm (OD_600_) of the culture in each well was measured at 10-min intervals. Before each measurement, the plate was briefly shaken to suspend the cells. Each experiment was performed on at least three independent biological replicates.

### RNA Isolation and Quantitative Reverse Transcription Polymerase Chain Reaction

RNA was isolated from 4 μl × 100 μl samples of *E. coli* K-12 cultures incubated for 6-h with sub-MIC concentrations of each respective antisense (KFF)_3_K-PNA oligomer (8 μM for anti-*mazE*, 4 μM for anti-*hipB*, 16 μM for anti-*thyA*, and 1 μM for anti-*gltX*). The cells were collected by centrifugation, and total bacterial RNA was isolated using a High Pure RNA Isolation Kit (Roche Molecular Systems), following the protocol provided by the manufacturer. All qPCR procedures were performed according to the MIQE guidelines (Bustin et al., 2009). To detect the expression of the *mazE, mazF, thyA, hipB, hipA*, and *gltX* genes, 50 ng of the total RNA was reverse transcribed to generate cDNA using a Maxima First Strand cDNA Synthesis Kit for RT-qPCR with dsDNase (Thermo Scientific^TM^), as outlined in the manufacturer’s protocol. qPCR was performed using SYBR^®^ Green Master Mix (Bio-Rad Laboratories Inc., United States) according to the supplied instructions. The level of the transcript of the essential *gyrA* gene was used for normalization (Rocha et al., 2015). The gene-specific primer pairs used for qPCR are listed in Supplementary Table 3. Amplification was performed in a Biorad CFX96 Touch^TM^ Real-Time PCR Detection System (Bio-Rad Laboratories Inc., United States). Relative quantification of gene transcription was performed using the ΔΔCT method (Livak and Schmittgen, 2001). The presented data represent averages of triplicate determinations, performed at least two times. Statistical analyses were performed with one-way ANOVA with Fisher’s test for multiple comparisons. Differences between treated and non-treated control samples were considered not significant (*P* ≥ 0.05), marginally significant (*P* < 0.05)^∗^, or significant (*P* < 0.01)^∗∗^.

### Synergy With Antibiotics – Fractional Inhibitory Concentrations

The interaction of (KFF)_3_K-PNAs and antibiotics was examined using a standard checkerboard microdilution method (Figure 5B; Hsieh et al., 1993). Samples of *E. coli* O157:H7 culture grown in MHB were added to the wells of sterile 96-well plates containing combinations of two agents at various concentrations, and the plates were incubated at 37°C for 12 h. The OD_600_ of the culture in each well was then determined using a plate reader. To define the nature of the interactions, the Fractional Inhibitory Concentration Indices (FICi) were calculated for each well with the equation FICi = FIC_A_ + FIC_B_ = (C_A_/MIC_A_) + (C_B_/MIC_B_), where MIC_A_ and MIC_B_ are the MICs of compounds A and B alone, respectively, and C_A_ and C_B_ are the concentrations of the compounds in combination, respectively. The FICi values were interpreted using a conservative model restricting interpretation to the following interaction categories: synergy (FICi ≤ 0.5), additive (FICi > 0.5–1.0), and indifference (1.0–4.0) (Doern, 2014).

### Cytotoxicity Assay

HEK-293 cells were cultured in DMEM medium with high glucose (Lonza) supplemented with 10% FBS (Biowest) and Penicillin–Streptomycin (50 U/ml, Gibco) at 37°C in 5% CO_2_. The cells were seeded at 5 × 10^4^ or 10^5^ per well in 96-well plates for incubations of 24 or 48 h, respectively. Wells contained the (KFF)_3_K-PNAs at a final concentration of 32 μM. Cell viability following incubation with the (KFF)_3_K-PNAs was determined using the standard MTT [3-(4,5-dimethylthiazol-2-yl)-2,5-diphenyltetrazolium bromide] (Merck) assay (Mosmann, 1983) with absorbance at 590 nm measured using a plate reading spectrophotometer (Synergy H1MFDG, Biotek). Each experiment was performed for three independent biological replicates. Statistical significance compared to untreated control samples was determined using the Mann-Whitney U test. All results were considered as statistically significant if *P* < 0.05.

## Data Availability Statement

The raw data supporting the conclusions of this manuscript will be made available by the authors, without undue reservation, to any qualified researcher.

## Author Contributions

MR designed the PNA sequences, performed the MIC, RT-qPCR, and FIC experiments, and analyzed the data. TP synthesized the PNA oligomers and PNA-peptides. JC, DB, and MR designed and prepared the *E. coli* K-12 MG1655 *ΔmazF* mutant. MK examined the cytotoxic effect of (KFF)_3_K-PNAs on eukaryotic cells. JT and DB supervised the overall project. MR, TP, and JT wrote the manuscript. All authors discussed the results and revised the manuscript.

## Conflict of Interest Statement

The authors declare that the research was conducted in the absence of any commercial or financial relationships that could be construed as a potential conflict of interest.
