# Supplementary material for: Artificial Activation of Escherichia coli mazEF and hipBA Toxin–Antitoxin Systems by Antisense Peptide Nucleic Acids as an Antibacterial Strategy
Source: Front Microbiol. 2018 Nov 26;9:2870. doi: 10.3389/fmicb.2018.02870 (PMC6275173; doi:10.3389/fmicb.2018.02870)
Supplement: Supplementary file 1 [file Data_Sheet_1.PDF]

Artificial activation of *Escherichia coli mazEF* and *hipBA* toxin-antitoxin systems by antisense peptide nucleic acids as an antibacterial strategy

Marcin Równicki[1,2], Tomasz Pieńko[1,3], Jakub Czarnecki[4,5], Monika Kolanowska[1,6] , Dariusz Bartosik[4], Joanna Trylska[1*]

[1]Centre of New Technologies, University of Warsaw, Banacha 2c, 02-097 Warsaw, Poland

[2]College of Inter-Faculty Individual Studies in Mathematics and Natural Sciences, University of Warsaw, Banacha 2c, 02-097 Warsaw, Poland

[3]Department of Drug Chemistry, Faculty of Pharmacy with the Laboratory Medicine Division, Medical University of Warsaw, Banacha 1a, 02-097 Warsaw, Poland

[4]Department of Bacterial Genetics, Institute of Microbiology, Faculty of Biology, University of Warsaw, Miecznikowa 1, 02-096, Warsaw, Poland

[5]Unit of Bacterial Genome Plasticity, Department of Genomes and Genetics, Institute Pasteur, 25-28 rue du Dr Roux, 75015 Paris, France

[6]Genomic Medicine, Medical University of Warsaw, Banacha 1a, 02-097 Warsaw, Poland

* **Correspondence:** Joanna Trylska: joanna@cent.uw.edu.pl

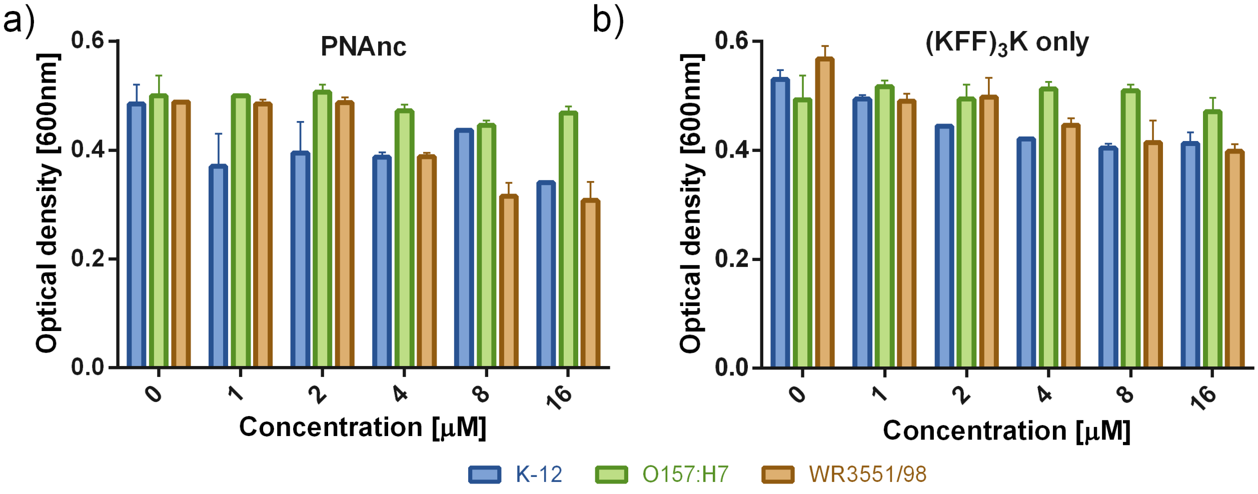

Supplementary Figure 1. No growth inhibition of *E. coli* strains after 20 h incubation with a) PNAnc and b) $(KFF)_3K$ peptide. Error bars represent the standard error of the mean of 3 replicates. The differences between corresponding samples are not significant (P > 0.05).

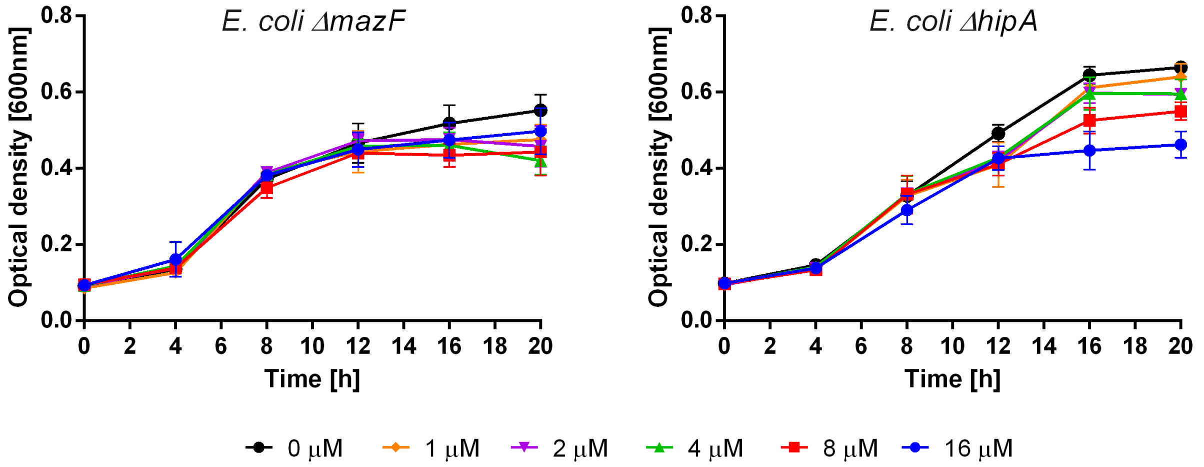

Supplementary Figure 2. No growth inhibition of *E. coli* strains lacking toxin genes after 20 h incubation with the corresponding PNAs. Error bars represent the standard error of the mean. The differences between 0 µM and 16 µM at 20 h for *E. coli ΔmazF* are not significant (P > 0.05) and for *E. coli ΔhipA* are significant (P < 0.01).

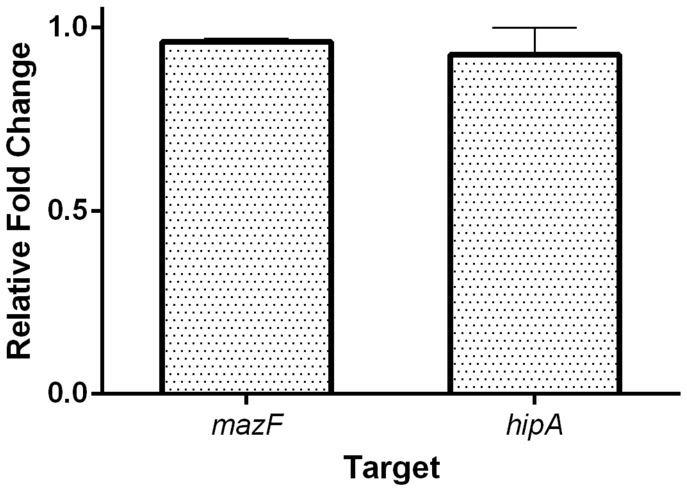

Supplementary Figure 3. The effect of treatment with PNAs on the level of toxin mRNA transcripts in *E. coli* K-12. The graph shows the relative fold change in expression compared to untreated control samples. Error bars represent the mean ± SEM, n=2.

Supplementary Table 1. Bacterial strains used in this study.

Strains	Relevant characteristics	Reference
E. coli K-12 MG1655	F^{-} λ^{-} wild-type strain	(Guyer et al., 1981)
E. coli 0157:H7 ST2-8624	(Δ*stx2::cat gfp)*	(Łoś et al., 2008)
E. coli WR3551/98	*bla*$_{CTX\text{-}M\text{-}15}$ ESBL+	(Baraniak, 2002)
E. coli DH5αλpir	F- *ΔlacU169 (ΦlacZΔM15), recA1, endA1, hsdR17, thi-1, gyrA96, relA1, λpir+*	(Platt et al., 2000)
E. coli β2163	MG1655::*ΔdapA::(erm-pir)RP4-2-Tc::Mu [Km*^{R}*]*	(Demarre et al., 2005)
E. coli K-12 MG1655 *ΔmazF*	MG1655 (*ΔmazF::kan)*	This study
E. coli JW1500-2 *ΔhipA*	*ΔhipA728::kan*	(Baba et al., 2006)

Supplementary Table 2. Oligonucleotide primers used in this study.

Name	Sequence (5' → 3')	Application
pds132X	CCCATGTCAGCCGTTAAGTG	construction of a cassette for deletion of *mazF*
pds132Y	ATCGCGCGGGTTTGTTACTG	
mazF1	CAGTAACAAACCCGCGCGATCTACTCCGCCG GATATTCGC	
mazF2	AGAGATTTTGAGACACAACGGATCGCCCATA TCGGGTACG	
mazF3	TAATTGGTTGTAACACTGGCAGAGGAGCAAC GAAGAAAGG	
mazF4	CACTTAACGGCTGACATGGGCAAGCGTCGTC CAATCGAAG	
KML	CGTTGTGTCTCAAAATCTCT	
KMR	GCCAGTGTTACAACCAATTA	
LmazFspr	CAAGCGTCGTCCAATCGAAG	confirmation of *mazF* deletion
RmazFspr	GGCGCGGTATTTCAGTACAC	
mazE_F1	AAGCGTTGGGGAAATTCACC	RT-qPCR for *mazE* transcript
mazE_R1	AATTTGCCATCCACCAGGTC	
mazF_F1	ACAAAAGGTAGCGAGCAAGC	RT-qPCR for *mazF* transcript
mazF_R1	GCGTTGTACAAGGAACACACAG	
thyA_F1	GGCCAGTGTATGGTAAACAG	RT-qPCR for *thyA* transcript
thyA_R1	TATCCAGTTCGCCTACGTTC	
hipB_F1	TTAAGCAGGCGACGATTTCC	RT-qPCR for *hipB* transcript
hipB_R1	TTCTGGCGAGGCATTTTTCG	
hipA_F1	TGGCGAAAGAACTTGGGTTG	RT-qPCR for *hipA* transcript
hipA_R1	AACGCCTGTCAAAACGTTCG	
gltX_F1	CGTTTTGATCGCTACAACGC	RT-qPCR for *gltX* transcript
gltX_R1	TTTCGCCATTTGCTCTTCGC	
gyrA_F1	TGGAAGTTGACGCCAAAACC	RT-qPCR for *gyrA* transcript (reference)
gyrA_F2	ATGCCTTCCACGCGTTTTTC	
mazEF-For	GCAGGCGCTGCATAATAGTG	amplification of *mazE* gene for DNA sequencing
mazEF-Rev	TGCGCCAGAACGCATTGTTG	

hipBA-For	GCTGTCTCGCCCTATTTCTG	amplification of *hipB* gene for DNA sequencing
hipBA-Rev	CGCCGTGAAGATGAATGGTC	
gltX-For	TAGAAACGTGCGCGTAAACC	amplification of *gltX* gene for DNA sequencing
gltX-Rev	ACGGCGGAGACTACATAAAG	
thyA-For	TGCTGCGCCATCATATGCAC	amplification of *thyA* gene for DNA sequencing
thyA-Rev	CGCACCACTCATTCCGTTTG	

Supplementary Table 3. Retention times (tR) and molecular masses of the synthesized conjugates. [a]The product was analyzed by RP-HPLC. To increase water solubility, each PNA has a lysine at the C-terminus.

					**Molecular mass [g/mol]**	
Nr	Compound	Short name	HPLC tR[a] [min]	HPLC method	**Calculated**	**Detected**
1	$(KFF)_3$K-AEEA-PNA anti-*mazE*-K	anti-*mazE* PNA	9.0	26-29% ACN 30 min	4833.17	4832
2	$(KFF)_3$K-AEEA-PNA anti-*thyA*-K	anti-*thyA* PNA	13.3	25-32% ACN 30 min	4880.17	4879.5
3	$(KFF)_3$K-AEEA-PNA anti-*hipB*-K	anti-*hipB* PNA	24.9	0-50% ACN 30 min	4646.87	4646.25
4	$(KFF)_3$K-AEEA-PNA anti-*gltX*-K	anti-*gltX* PNA	20.7	0-50% ACN 30 min	4401.67	4401.5
5	$(KFF)_3$K-AEEA-PNAnc-K	PNAnc	12.7	25-35% ACN 30 min	4880.17	4879.5

(KFF)$_3$K-AEEA-PNA anti-*mazE*-K

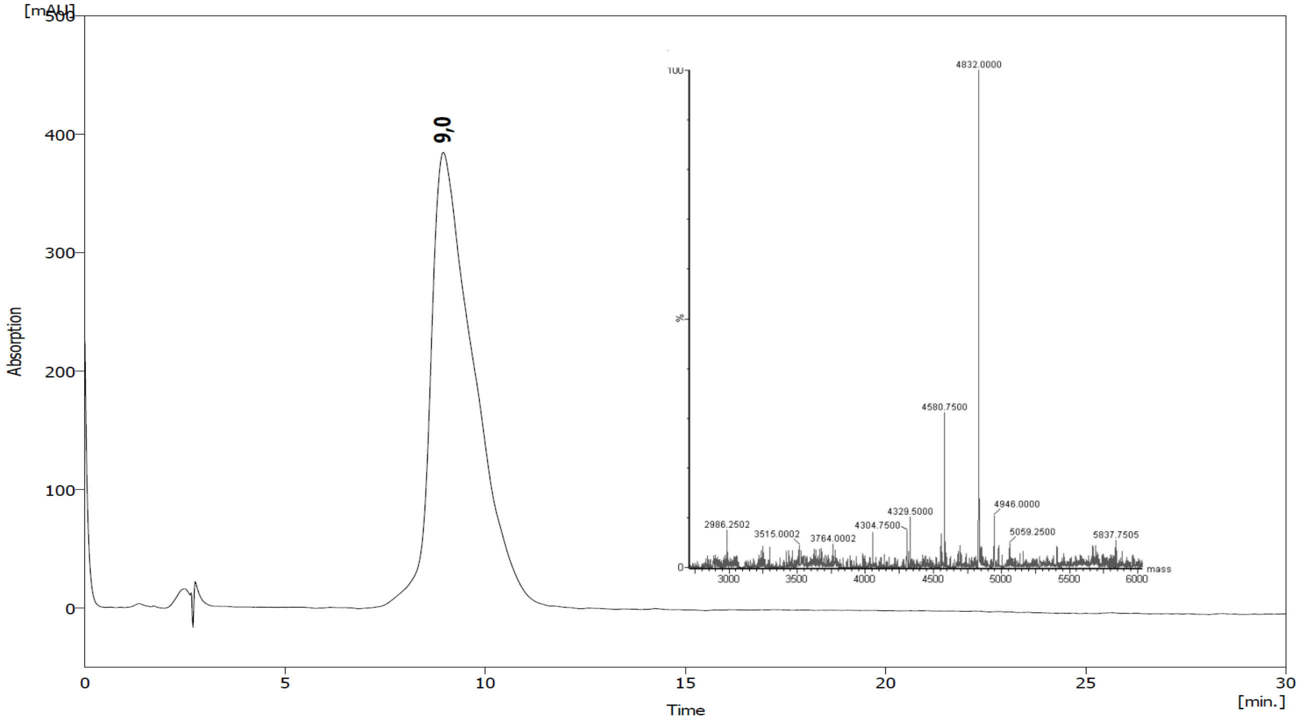

Supplementary Figure 4. Mass spectrum and HPLC chromatogram for (KFF)$_3$K-AEEA-PNA anti-*mazE*-K

(KFF)$_3$K-AEEA-PNA anti-*thyA*-K

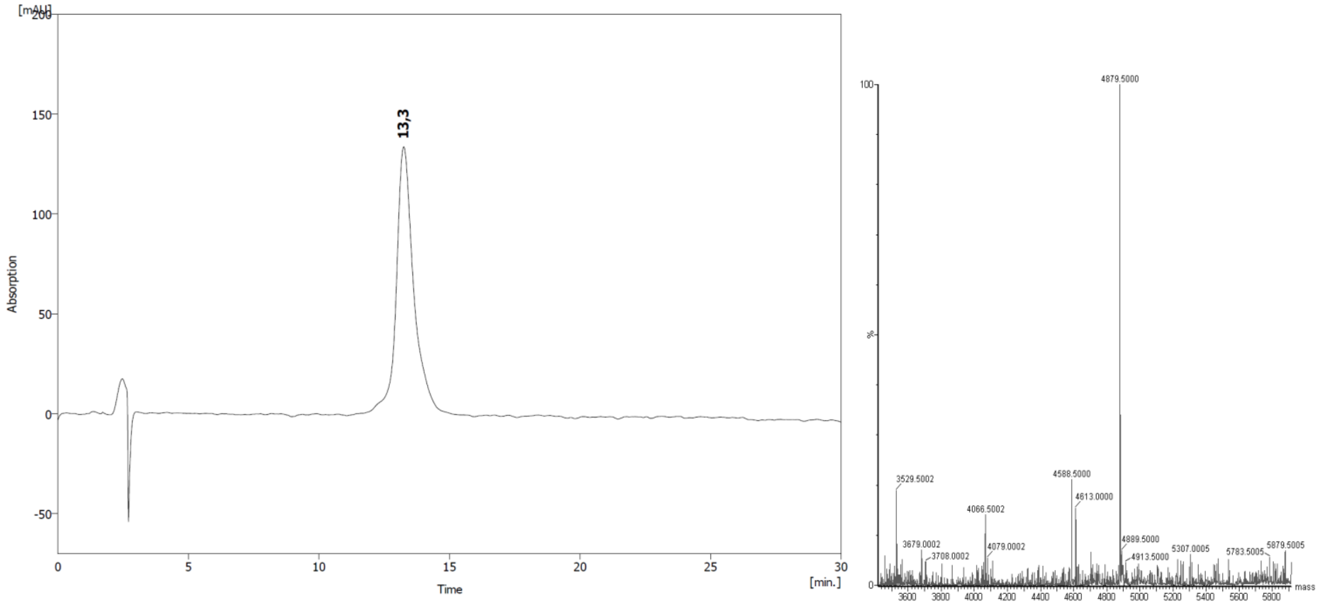

Supplementary Figure 5. Mass spectrum and HPLC chromatogram for (KFF)$_3$K-AEEA-PNA anti-*thyA*-K

(KFF)$_3$K-AEEA-PNA anti-*hipB*-K

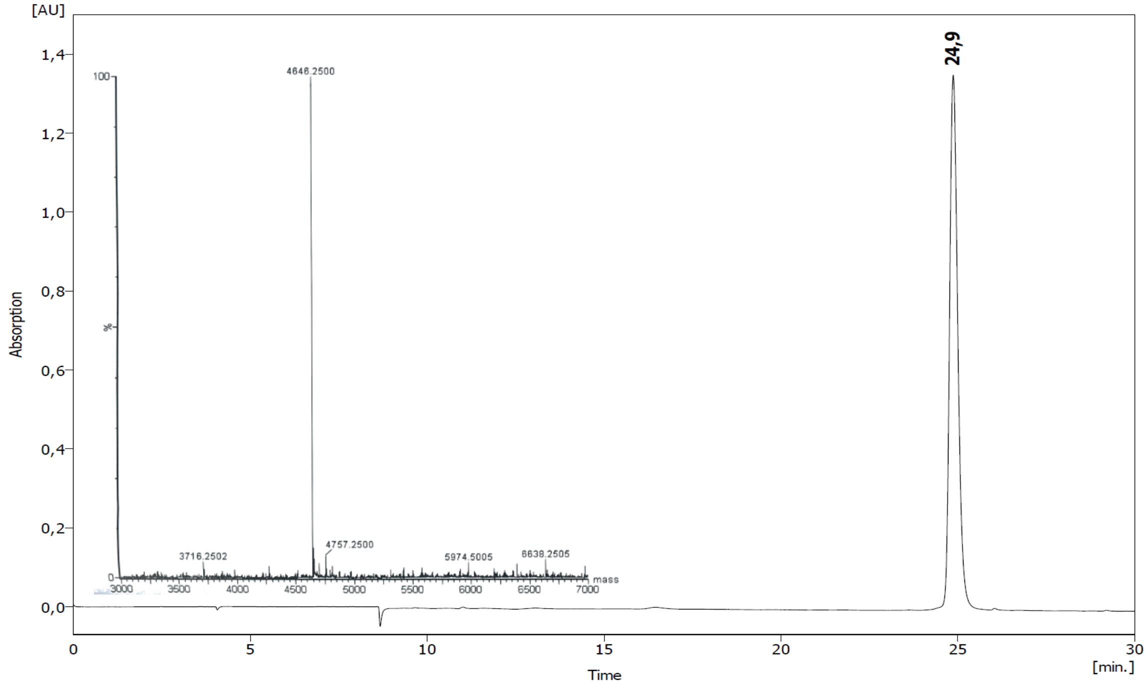

Supplementary Figure 6. Mass spectrum and HPLC chromatogram for (KFF)$_3$K-AEEA-PNA anti-*hipB*-K

(KFF)$_3$K-AEEA-PNA anti-*gltX*-K

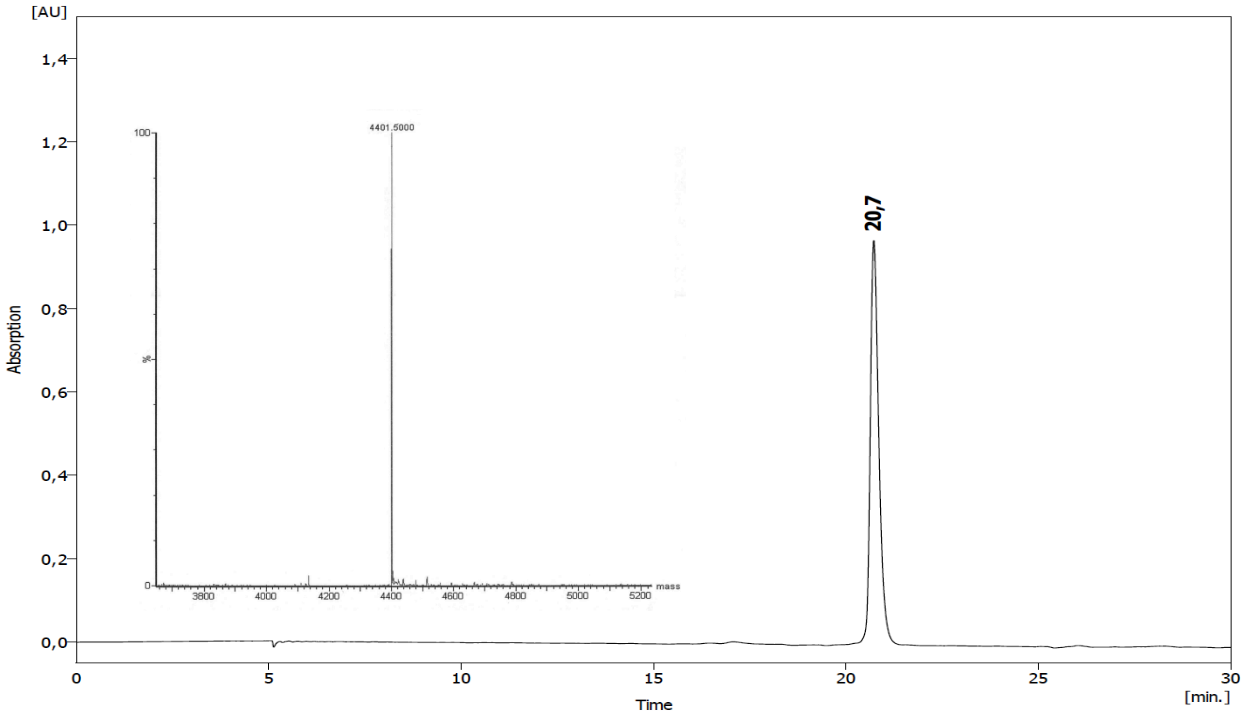

Supplementary Figure 7. Mass spectrum and HPLC chromatogram for (KFF)$_3$K-AEEA-PNA anti-*gltX*-K

(KFF)$_3$K-AEEA-PNAnc-K

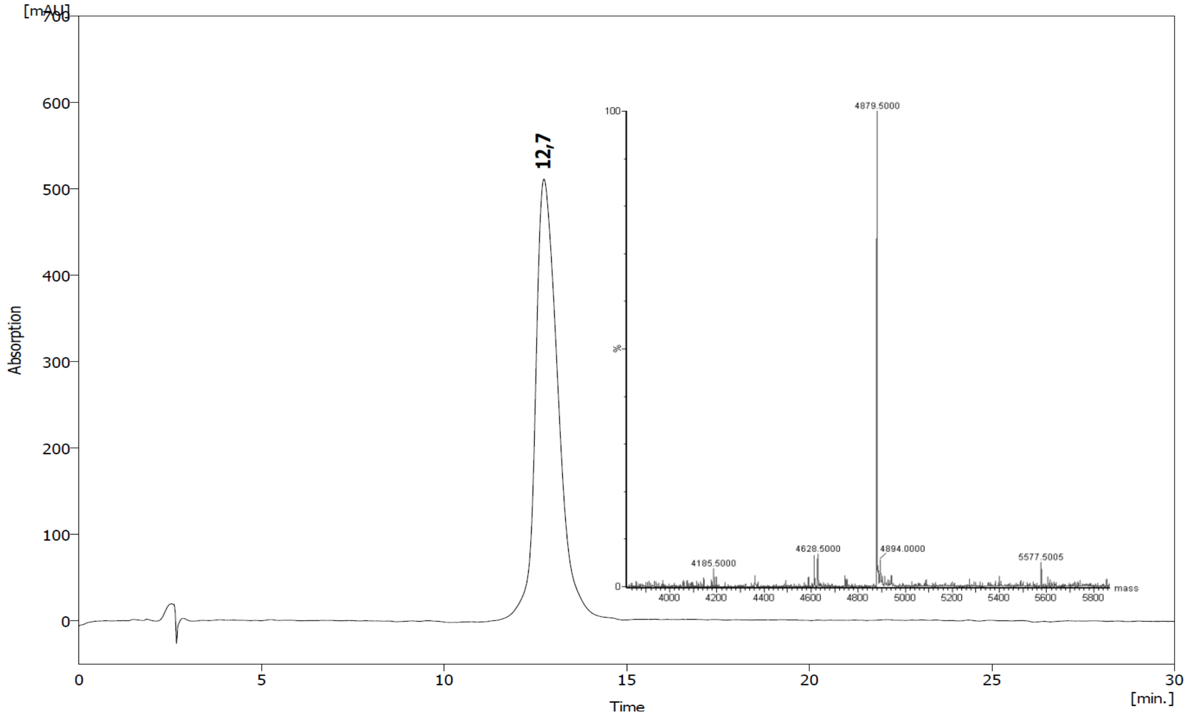

Supplementary Figure 8. Mass spectrum and HPLC chromatogram for (KFF)$_3$K-AEEA-PNAnc-K

References:

Baba, T., Ara, T., Hasegawa, M., Takai, Y., Okumura, Y., Baba, M., et al. (2006). Construction of Escherichia coli K-12 in-frame, single-gene knockout mutants: The Keio collection. *Mol. Syst. Biol.* 2. doi:10.1038/msb4100050.

Baraniak, A. (2002). Ceftazidime-hydrolysing CTX-M-15 extended-spectrum beta-lactamase (ESBL) in Poland. *J. Antimicrob. Chemother.* 50, 393–396. doi:10.1093/jac/dkf151.

Demarre, G., Guérout, A. M., Matsumoto-Mashimo, C., Rowe-Magnus, D. A., Marlière, P., and Mazel, D. (2005). A new family of mobilizable suicide plasmids based on broad host range R388 plasmid (IncW) and RP4 plasmid (IncPα) conjugative machineries and their cognate Escherichia coli host strains. *Res. Microbiol.* 156, 245–255. doi:10.1016/j.resmic.2004.09.007.

Guyer, M. S., Reed, R. R., Steitz, J. A., and Low, K. B. (1981). Identification of a sex-factor-affinity site in E. coli as gamma delta. *Cold Spring Harb. Symp. Quant. Biol.* 45, 135–140. doi:10.1101/SQB.1981.045.01.022.

Łoś, J. M., Golec, P., Węgrzyn, G., Węgrzyn, A., and Łoś, M. (2008). Simple method for plating Escherichia coli bacteriophages forming very small plaques or no plaques under standard conditions. *Appl. Environ. Microbiol.* 74, 5113–5120. doi:10.1128/AEM.00306-08.

Platt, R., Drescher, C., Park, S. K., and Phillips, G. J. (2000). Genetic system for reversible integration of DNA constructs and lacZ gene fusions into the Escherichia coli chromosome. *Plasmid* 43, 12–23. doi:10.1006/plas.1999.1433.
